# Do Machines Replicate Humans? Toward a Unified Understanding of Radicalizing Content on the Open Social Web

**DOI:** 10.1002/poi3.223

**Published:** 2019-09-26

**Authors:** Margeret Hall, Michael Logan, Gina S. Ligon, Douglas C. Derrick

**Keywords:** social media, violent extremist organizations, LIWC, latent dirichlet allocation, n‐grams, counter terrorism

## Abstract

The advent of the Internet inadvertently augmented the functioning and success of violent extremist organizations. Terrorist organizations like the Islamic State in Iraq and Syria (ISIS) use the Internet to project their message to a global audience. The majority of research and practice on web‐based terrorist propaganda uses human coders to classify content, raising serious concerns such as burnout, mental stress, and reliability of the coded data. More recently, technology platforms and researchers have started to examine the online content using automated classification procedures. However, there are questions about the robustness of automated procedures, given insufficient research comparing and contextualizing the difference between human and machine coding. This article compares output of three text analytics packages with that of human coders on a sample of one hundred nonindexed web pages associated with ISIS. We find that prevalent topics (e.g., holy war) are accurately detected by the three packages whereas nuanced concepts (Lone Wolf attacks) are generally missed. Our findings suggest that naïve approaches of standard applications do not approximate human understanding, and therefore consumption, of radicalizing content. Before radicalizing content can be automatically detected, we need a closer approximation to human understanding.

## Abstract (zh)

互联网的出现增强了暴力极端主义机构的成功运作。伊斯兰国（ISIS）等恐怖主义机构利用互联网将其企图传递的信息投射给全球受众。针对网络恐怖主义宣传的大多数研究和实践都使用人工编码员来对内容进行分类，这引起了有关工作倦怠、精神压力、编码数据可信度的严重忧虑。近期，技术平台和研究者已开始使用自动分类程序来检验网络内容。然而，考虑到有关将人工编码和机器编码进行比较、以及将二者的不同之处放入情境中加以理解的研究并不充足，自动程序的健全性存在疑问。针对一项包含100个与ISIS相关的非索引网页的样本，本文比较了三种文本分析程序和人工编码分别得出的结果。作者发现，三种程序精确地识别出了普遍话题（例如圣战等），然而却基本没有识别出微妙的概念（孤狼恐怖袭击）。研究结果表明，标准化程序这一简单措施无法赶上人类思维理解，因此也无法处理极端化内容。在极端化内容能被自动检测出之前，（自动程序）需要更接近人类思维理解。

## Abstract (es)

La llegada de Internet ha aumentado, sin darse cuenta, el funcionamiento y el éxito de las organizaciones extremistas violentas. Las organizaciones terroristas como el Estado Islámico de Irak y Siria (EI) utilizan el Internet para proyectar sus mensajes a una audiencia mundial. La mayoría de las investigaciones y prácticas sobre propaganda terrorista basada en la web utilizan codificadores humanos para clasificar el contenido, lo que plantea serias preocupaciones como el agotamiento, el estrés mental y la confiabilidad de los datos codificados. Más recientemente, las plataformas de tecnología y los investigadores han empezado a examinar el contenido en línea a través del uso de procedimientos de clasificación automatizadas. Sin embargo, hay preguntas acerca de qué tan robustos son los procedimientos automáticos, dada la insuficiente investigación que compare y contextualice la diferencia entre la codificación humana y la automática. Este artículo compara el resultado de tres paquetes de análisis de texto con el de los codificadores humanos en una muestra de 100 páginas web no indexadas asociadas con ISIS. Hallamos que los temas prevalentes (por ejemplo: guerra santa) son exitosamente detectados por los tres paquetes, mientras que los conceptos más matizados (ataques de lobo solitario) no lo son. Nuestros hallazgos sugieren que los métodos ingenuos de aplicaciones estándar no se acercan a la comprensión humana, y por ende al consumo, del contenido radicalizador. Antes de que el contenido radicalizador pueda ser detectado automáticamente, necesitamos una aproximación más cercana a la comprensión humana.

## Introduction

The world is seemingly continually surprised by the advance of deviant content on social media and the open Internet (Ingram, 2016). This is particularly true in the case of violent extremist organizations (VEOs)—that is, coordinated efforts among individuals with a shared ideological framework and goal toward collective actions (Ligon, Simi, Harms, & Harris, 2013). The capacity for posting and hosting ever‐more disturbing, violent propaganda is matched only by the willingness of (vulnerable) individuals to consume it. Attempting to proactively detect (and remove) VEO content from open social platforms is a large‐scale social and research problem (UNCTED, 2016, Whine, 2010).

As VEO content appears online it swims in an ocean of other unrelated content, leaving the noise‐to‐signal ratio very high. To solve the detection problem, manual approaches like content coding, or labeling, have been standard. On one hand, manual approaches have proven to be inappropriate to the task of identifying and treating content at scale. Human understanding is the “gold standard” of classification, but with respect to VEO content it is a poor strategy due to aspects like coder burnout and potential errors and bias due to inexact classification definitions. Especially, with nuanced or latent VEO content there can be worldview conflicts (e.g., conflicts with personally held beliefs on free speech or religion) leading to stress in the coder, and few explicit, standardized definitions that apply to online material exist except in cases of depictions of crime (Facebook, 2019; Logan & Hall, 2019). Even with these potential downsides, manual coding is currently in use, or forms part of an artificial intelligence (AI) strategy at the major social content platforms and research labs (Alonso, 2019). On the other hand, even the most successful AI tools misclassify radicalizing content. For example, Facebook reported finding 99 percent of terror‐related content in Q1‐3 of 2018 (https://newsroom.fb.com/news/2018/11/staying‐ahead‐of‐terrorists/). This leaves an estimated one hundred forty‐three thousand terror‐related content items, which were unable to be classified. The length of time the identified items were available on the platform before being identified ranged from two minutes to seven hundred and seventy days in Q3 2018. Most misclassification comes from the advent of new modalities of content provision or from disagreement between human definitions and machine classification. These misclassifications suggest a potential systematic bias in the classifier in use.

Given its positive attributes, including increased efficiency and low researcher exposure, automated detection of VEO content online should be a broad research and societal goal (Correa & Sureka, 2013). However, a fundamental challenge is reducing the barriers to machine understanding (Alonso, 2019), given even in binary classification schemes some measure of disagreement exists (Hashemi & Hall, 2018, 2019). Approaches to classification tend to either be fully machine (e.g., Alghamdi & Selamat, 2012; Sabbah, Selamat, & Selamat, 2014; Scrivens, Davies, Frank, & Mei, 2016) or human (e.g., Droogan & Peattie, 2016; Ligon, Hall, & Braun, 2018; Torres, Jordán, & Horsburgh, 2006; Torres‐Soriano, 2016). Very few studies to date compare performance of humans and machines when classifying the underlying themes of radicalizing content. Even those with mixed modalities rarely show empirically the degree to which human coders and machines classifying the same radicalizing content have similar results (Cohen, Kruglanski, Gelfand, Webber, & Gunaratna, 2016; Correa & Sureka, 2013, Prentice, Taylor, Rayson, Hoskins, & O’Loughlin, 2011)—implying that we have an incomplete understanding of the degree of overlap of human and machine understandings of content and its classification. This uncertainty has an unknown impact on the way stakeholders manage their detection/classification regimes. Each of these differences contributes to/against external and internal validity and overall reliability of the analyses (Ruths & Pfeffer, 2014). Applications and algorithms to be deployed in the future are dependent upon some agreement of which content is radical: this does not currently exist.

We propose to help close that gap by considering the domain of text analysis. This study applies two probabilistic topic modeling algorithms and one off‐the‐shelf sentiment analysis package to an extant data set, namely 100 transient websites associated with the Islamic State (ISIS) (see the Methodology section). The coding themes in use are created by human coders evaluating ISIS content from these one hundred open websites (Derrick, Sporer, Church, & Ligon, 2017b). We introduce the themes established in (Derrick et al., 2017b) and refer to this throughout as the “benchmark study.” The current study identified the probabilistic topic models n‐grams (Jurafsky & Martin, 2014) and latent dirichlet allocation (LDA) (Blei, Ng, & Jordan, 2003) as the most appropriate, albeit simplistic, methodologies to assess the unlabeled corpus for a first attempt at comparing the performance of human and machines when coding VEO content. In addition, we assess the results of the Linguistic Inquiry and Word Count (LIWC) sentiment analysis program (Pennebaker, 2011). We chose these three packages due to their prevalence in related studies, their relatively strong benchmarks, and the ease of use for noncomputational scholars. The aim of this evaluation is assessing *the degree to which computer‐mediated coding and human coders agree on the underlying themes of a radical corpus*. The thematic view in use reflects the reality that different propaganda types exist, and that these differently impact individuals’ intent to act offline (Crosset, Tanner, & Campana, 2019).

It is important to identify the degree to which a gap exists between humans and machines due to the nature of the content. The imperative from a platform perspective reflects that VEO content generally violates their Terms of Service. More important is the human dimension: individuals consuming VEO content on the open social web may be vulnerable to its messaging. Pathways to radicalizing are many (Ligon et al., 2018); appropriately diagnosing radicalizing content before it can impact an individual is therefore critical. Finding that machines and humans are reasonably similar in their understanding of the themes that exist in radicalizing content would support a stronger content management regime overall.

In the next section we present and classify the relevant literature, showing that the question of performance of automated algorithms compared with human‐coded benchmarks in VEO content is under‐addressed in the literature. We present our research question, and then introduce the experimental design, data, and methodology. We then evaluate the results, and discuss their implications. We conclude with suggestions for expanding the research, and note some limitations to our approach.

## Usage Patterns of VEOs Online

The promulgation of open‐ and free‐Internet architectures requires less technical infrastructure for smaller or resource‐poor organizations to communicate and conduct operations (Derrick, Ligon, Harms, & Mahoney, 2017a). Asymmetric organizations such as VEOs and criminal groups likewise expanded their reach and capacity for operations. VEOs have narrowed the infrastructure gap with larger, more affluent adversaries who have the means to develop expensive infrastructure for internal communication (e.g., the US Government invests in internal communication portals for secure communication). VEOs leverage existing (Internet) technologies to conduct their operations, recruit members, solicit financing, and facilitate strategic objectives during conflict (Denning, 2010; Weimann, 2004). The lowering of technological barriers has changed organizations’ behaviors on the short‐end of the power asymmetry and enabled the historically disadvantaged VEOs to launch social media campaigns akin to those of large governments and corporations (Balmer, 2001; Bartlett and Reynolds, 2015; Fenstermacher & Leventhal, 2011).

Given the web’s inherent anonymity, limited regulation, rapid flow of information, and large audience (Weimann, 2004), the Internet has prompted a shift in criminal and deviant behavior. In a review of how the Internet may amplify deviant communities online (Recupero, 2008), defined and examined the online disinhibition effect of fifty‐three groups including propedophilia, hate groups, proeating disorders groups, and terrorist groups. She found that problematic internet use by individuals interested in these groups increased dramatically. Relatedly, clandestine digital forums allow participants to express deviant opinions with little fear of negative social consequences, such as being discredited or stigmatized by mainstream elements (Steinfeldt et al., 2010).

Much research has been conducted on online behaviors of deviant groups, such as the far right and racially‐motivated groups (Caren, Jowers, & Gaby, 2012; Holt & Bolden, 2014; Steinfeldt et al., 2010), animal‐rights extremists (Braddock, 2015), gangs and gang members (Chen et al., 2004; Pyrooz, Decker, & Moule, 2013), proanorexia support groups (Boero & Pascoe, 2012; Fox, Ward, & O’Rourke, 2005), sex crimes and human trafficking (Dubrawski, Miller, Barnes, Boecking, & Kennedy, 2015; Elliott & Beech, 2009; Latonero, 2011; Nagpal, Miller, Boecking, & Dubrawski, 2017), and the drug trade (Fox, Ward, & O’Rourke, 2006; Martin, 2013). VEOs’ Internet usage is similar to other types of online criminal activities (Recupero, 2008) in that it is used to disseminate information and acquire value resources such as recruits, funds, and weapons. For example, Moule, Pyrooz, and Decker (2014) show that street gangs with a high degree of organization are more likely to utilize the Internet for recruitment and branding relative to other street gangs. It must be noted, however, that while some studies on deviant groups work in the automated detection space (e.g., Dubrawski et al., 2015), the characteristics of the signals from each type of deviant group are quite distinct—signals and detection benchmarks of child pornography, gangs, or supremacists do not necessarily transfer to jihadi content. The importance of our ability to quickly and reliably detect and classify radicalizing content became apparent in the aftermath of the 2019 Christchurch mosque shootings: a live feed of the attack from the assailant’s bodycam was taken down from all large social media platforms where it was hosted. The platforms, however, did not take down the feed before it was copied, sometimes recut, and reposted hundreds of thousands of times across platforms like Facebook, Reddit, and Twitter (https://www.washingtonpost.com/technology/2019/03/18/inside‐youtubes‐struggles‐shut‐down‐video‐new‐zealand‐shooting‐humans‐who‐outsmarted‐its‐systems/?utm_term = .bb581c44ea23). YouTube was forced to restrict search functionality as the reposting of the (sometimes remastered) video overwhelmed their algorithmic approach.

### Recruiting

One of the most interesting aspects of VEOs is their use of information and communication technologies (ICTs) to recruit members and acquire skills. Recruiting is a central component of VEO leader decision making, resulting in a focus on branding, organizational legitimacy, and creating a compelling narrative.^1^ Decisions are both made and framed in relation to the brand, such as what alliances to endorse, what media to use in recruiting, and what statements to make by key figures. In the late 1990s and early 2000s, for example, members of the Chechen Mujahidin would film and distribute their armed attacks on the Russians on the Internet.^2^ While the Chechen militants were aware of the marginal effects of their attacks, they were also cognizant that hosting these attacks online would have greater effects in terms of mobilizing sympathizers of their cause and demoralizing the opposition (Torres et al., 2006). Additionally, VEOs utilize the Internet to establish online or virtual communities of sympathizers and supporters. Individuals who feel otherwise disconnected may gravitate toward virtual communities where they feel accepted. As such, virtual communities are a way for VEOs to foster a sense of in‐group membership and reach potential recruits in a way that could not be reached through traditional face‐to‐face recruitment (Radlauer, 2007; Simi & Futrell, 2006).

As previously noted, VEOs are strategic about what media they publish online. VEOs, like any organization, are concerned about establishing and maintaining their brand given its influence on their target audiences’ (friend and foe) perceptions about the group (Pelletier, Lundmark, Gardner, Ligon, & Kilinc, 2016). In turn, VEOs engage in innovative marketing strategies and craft specific types of media to draw potential recruits toward the movement. For instance, Al Qaeda and its regional affiliates (e.g., AQIM and AQAP) frequently craft messages that highlight the ongoing “battle of civilizations” and the struggle against the far enemy (United States). Al Qaeda deliberately used this narrative to project themselves as a global force, appealing to bored and alienated young males with the promise of excitement and adventure (Baines & O’Shaughnessy, 2014; Baines et al., 2010). Alongside violent narratives, VEOs such as ISIS have also capitalized on using non‐violent themes as a recruitment tool. During a month‐long period in 2015, Winter (2015) discovered that 52.57 percent of all online media outputs from ISIS emphasized utopian themes (also see Derrick et al., 2017b). These utopian themes focused on everyday life in the caliphate such as religious and social gatherings, economic activity, nature and wildlife, and governance. These themes are intended to promote ISIS and its caliphate as a legitimate state (Winter, 2015).

### Command and Control and Decision Making

VEOs use digital communications for command and control (Whine, 2010). This is especially true for deterritorialized and/or decentralized organizations where the Internet works as a command and control center to coordinate and dispersed membership or attacks. For example, ISIS has been particularly adept at expanding its organizational reach through the use of encrypted messaging applications. Recent evidence indicates that ISIS remotely organized and instructed a cell in India to carry out multiple attacks. Shortly after arresting five members of the cell, Indian authorities discovered that ISIS had provided the cell with weapons, ammunition, chemicals, and operational and technical assistance (Callimachi, 2017).

Previous work found that decision making occurs in private/deep web Internet technologies and/or in face‐to‐face interactions (Derrick et al., 2017b), with the open internet used to disseminate commands and to get reports on outcomes. The dissemination of the decisions and command and control instructions may be delivered in several ways. For example, Al Qaeda relays messages to the public or specific target audiences, and once an effective message is released, the public and news sources tend to spread the message with little effort from the group (Thomas, 2003).

### Transferring Ideological and Pragmatic Knowledge

Another way VEOs use ICT is to educate their followers. Essentially, ICT allows for the widespread transferring of knowledge among all types of followers. Knowledge transfer involves transmitting or sending knowledge to a potential recipient and the absorption or use of the transferred knowledge (Davenport, Delong, & Beers, 1998). VEOs have leveraged different ICTs such as knowledge repositories to support the transfer of knowledge. These repositories are especially relevant as they enable the rapid sharing of “know‐how” in an asynchronous manner. This know‐how is represented in two dominant themes, ideology, and pragmatism. First, VEOs use ICTs to transmit knowledge around “ideological purity” and vision. In other words, organizations use technology to communicate sacred values. A sacred value is more than just a shared value or moral value. It is a value that is prescribed by deity and by which adherents judge and evaluate their actions and ultimately their lives. Ideological leadership represents vision‐based leadership, where the vision is a compelling, emotionally evocative view that appeals to virtues of a past ideal state (e.g., a caliphate). Ideological leaders frame this vision around values and standards that must be maintained to rebuild this “pure” society (Mumford, Strange, & Bedell‐Avers, 2006). For example, VEOs use ICTs to identify and highlight violations of standards, morals, and codes of conduct that underlie the overarching ideology. Extreme ideological leaders tend to view violations of such standards and values in dichotomous terms, drawing distinct differences between adherents and nonadherents. The ability to identify and enumerate violations gives a sense of moral superiority and “justness” to the leaders of the cause. Their perceived and perpetuated rightness is necessary to build ideological legitimacy. The strict adherence removes dissonance that brings follower attitudes more in‐line with the leaders’ desires. Similarly, ideological leaders tend to hearken back to times of past greatness, drawing from key figures in the ideology and lessons learned from them. Ideological leadership compares present and past conditions. It can motivate and give a sense of time and purpose (Freeman, 2014; Hofmann, 2017; Hofmann & Dawson, 2014; Mumford et al., 2007).

Second, VEOs use ICTs for the transmission and application of both declarative and procedural knowledge through different presentation modes. These knowledge repositories are likely to be found in the deep web, but some are also freely available. There are sites that offer information about systems being used at locations of interest. Many of these knowledge repositories allow a user to enter system information for the desired target and find specific vulnerabilities that have been cited about that system or software. VEOs can use internet technologies to disseminate and train individuals virtually (Derrick et al., 2017b). This requires fewer experts and overcomes concern about colocation. This distance learning will be in private venues, possibly taking the form of synchronous or asynchronous packages.

## Existing Approaches

As illustrated above, VEOs across ideological types (e.g., religious, ethno‐nationalist, and left‐wing) use ICTs to further their ideological and organization goals. The remainder of this section, however, focuses on prior research on jihadist VEOs for two reasons. First, data for this study comes from ISIS—a VEO with jihadi motivations. Second, the past two decades have seen more and more interest in researching digital jihadist narratives. For example, Table 1 summarizes some of the recent works in this area. Although Table 1 is not an exhaustive list, it provides context for differentiation of this work to other approaches. Immediately noticeable is the preponderance of qualitative works using human raters in the VEO space. These “expert‐based” and/or “content‐analytic” studies rely on one or more human raters to identify and analyze different forms of ideological propaganda (Cohen et al., 2016, pp. 2–3). The common criticism of this approach is that it relies on subjective ratings and is open to biases. However, others suggest this approach is better at contextualizing information compared to automated techniques (Cohen et al., 2016). It is very clear that there are many potential negative downstream effects like burnout or post‐traumatic stress for those who repetitively view and process this content.

**Table 1:** Sample of Recent Works on VEO Use of Internet Technologies

Article	VEO(s)	Modality	Sample	Evaluation
Abrahms, Beauchamp, and Mroszczyk (2016)	Various Jihadi Groups (i.e., Al Qaeda in the Arabian Peninsula, Al Qaeda in the Islamic Maghreb, al‐Shabaab, Al Qaeda in Iraq, and Afghan Taliban)	Video	473 Videos *IntelCenter* database (Originally 900 videos)	Human
Baines and O’Shaughnessy (2014)	Al Qaeda Central	Video	37 Videos (from 97 videos) *IntelCenter* database	Human
Cohen et al. (2016)	Al Qaeda Central & Al Qaeda in Iraq	Text	18 Transcripts (from Site Intel & START organization)	Automated and Human
Droogan and Peattie (2016)	Al Qaeda in the Arabian Peninsula	Text	14 Issues of AQAP’s *Inspire* magazine	Human
Hafez (2007)	Various Jihadi Groups	Text, Image, and Video	29 Video clips, 5 audio clips, and over 20 online magazines	Human
Ingram (2016)	Al Qaeda in the Arabian Peninsula & Islamic State in Iraq and Syria (ISIS)	Text	14 issues of AQAP’s *Inspire* and 13 issues of ISIS’s *Dabiq Magazine*	Human
Klausen (2015)	Various Jihadi Groups	Text	563 Tweets from 59 social media accounts	Human
O’Halloran et al. (2016)	ISIS	Text and Image	*Dabiq* magazine	Automated
Pennebaker (2011)	Al Qaeda Central & Al Qaeda in the Arabian Peninsula	Text	296 Speeches, interviews, and articles	Automated
Prentice et al. (2011)	Various Jihadi Groups	Text	50 Online texts	Human and Automated
Qin, Zhou, Reid, Lai, and Chen (2007)	Various Jihadi Groups	Text, Image, and Video	222,000 Web documents	Automated
Smith, Suedfeld, Conway, and Winter (2008)	Al Qaeda Central & Al Qaeda in the Arabian Peninsula	Text	53 Speeches, interviews, and articles from original and current Al Qaeda Central leaders;	Human
			49 Documents signed by or issued by Al Qaeda in the Arabian Peninsula members	
Torres Soriano (2010)	Various Jihadi Groups	Text and Video	199 Documents	Human
Torres et al. (2006)	Various Jihadi Groups	Text, Image, and Video	2,878 Documents	Human
Torres‐Soriano (2016)	Al Qaeda in the Islamic Maghreb	Text	482 communiqués issued by AQIM between 1998‐2015	Human
Winter (2015)	ISIS	Text, Image, and Video	896 (1,146 total gathered) propaganda events	Human

*Note*: We did not “audio” as a separate modality because this generally goes hand‐in‐hand with the “video” classification*.*

A second interesting takeaway from Table 1 is the diverse number of modalities used to examine radicalizing propaganda. For example, several studies have included more than one modality to examine propaganda‐related narratives. This may be due to an increased awareness in the research community regarding the number and types of social media websites currently available to terrorist organizations. As Correa and Sureka (2013) suggest, deviant groups now have access to various communication modalities such as weblogs, online forums, image sharing websites (e.g., Flickr), and video sharing websites (e.g., YouTube). Thus, researchers’ ability to triangulate information sources is more important now than ever before. The ability to analyze multimodel content is further supported by the domain’s general reliance on qualitative approaches. Very few studies tie multiple platforms together (Hall, Mazarakis, Chorley, & Caton, 2018), and even fewer analyze multiple contents types and/or formats (Wendlandt, Mihalcea, Boyd, & Pennebaker, 2017).

Finally, Table 1 indicates that most of the recent works on Jihadi propaganda have focused on Al Qaeda and their affiliated movements (AQAM) and, to a lesser extent, ISIS. This is not surprising for two (related) reasons. First, AQAM and ISIS have dominated the Jihadi extremist market for several years now (Ligon et al., 2017). In turn, AQAM and ISIS are two of the most recognizable extremist “brands” with the capacity and legitimacy to produce extended propaganda campaigns. In other words, AQAM and ISIS can afford to publish extensive amounts of propaganda, while smaller and lesser‐known groups either completely lack resources or must funnel them to more pertinent organizational processes to survive.

### Limitations of Existing Approaches

As alluded to above, the primary limitation of earlier attempts to categorize types of messaging in extremist content is the overreliance on human coders. This is problematic for three reasons. First, the scalability of such approaches is minimal. The use of human coders is highly labor intensive. Extremist propaganda can also be horrifically violent and likewise disturbing to human coders or platforms’ content reviewers—for example, the footage of the 2015 immolation of a Jordanian pilot by ISIS. Burnout of the human coders is a real concern. Second, human coding strategies are susceptible to pre‐existing assumptions or biases held by the raters. In other words, the replicability and reliability of human‐coding strategies are limited. Third, although the use of computerized approaches has grown in recent years, few studies have examined the degree to computational methods compare with the benchmark solutions found by human coders when categorizing extremist content. In radicalization studies, often either human coders or computational pattern assessments are presented as holistic results. However, to our knowledge no work addresses if and to what extent a gap between human and machine understanding of radicalizing content exists. On the basis of these limitations, we present the guiding research question:

RQ: To what degree do computerized text‐analysis approximate benchmark solutions of human coders taken from extremist content on the open web?

## Methodology

### Data and Its Collection

**Figure 1:**
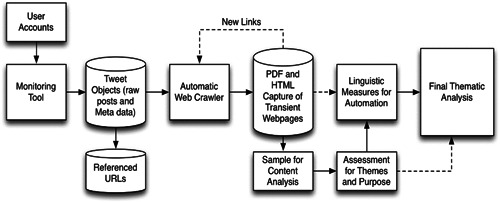
Flow Diagram for Monitoring and Scraping Transient Web Pages.

The data used in this exploratory study are one hundred English language nonindexed, transient web pages posted by ISIS activists or supporters to open, web‐based architectures. These data were harvested using a custom program extracting transient websites introduced and used in Derrick et al. (2017b) (Figure 1).

A faction of the hacktivist group Anonymous code‐named Controlling Section (@CtrlSec) was formed in early 2015 to help find and remove ISIS accounts from Twitter (Macri, 2015). During much of this collection, CtrlSec posted ISIS members’ Twitter handles at a rate of approximately one every two minutes. Using the Twitter API, the custom program (Figure 1) follows and logs the individual tweets dispatched by CtrlSec accounts. The program first started collecting accounts released by CtrlSec in August 2015 and has been running since. Using the handles posted by CtrlSec, the handles of suspected ISIS accounts are identified and followed using the Twitter API.

From that list, the system utilizes the Twitter API to download a sample of the latest tweets from the ISIS‐affiliated accounts. After being logged in the database, the tweets are sorted into metadata (e.g., number of web addresses and links, hashtags, mentions) and content (tweet text, links). Links which are posted in the tweets of ISIS accounts are used as launching points to the open architectures where richer content is housed. The software searches for links within tweets referencing anonymous posting services for open content‐publishing transient web pages (e.g., JustPaste.it, dump.to). Next, the software automatically crawls to the referenced webpage, captures PDF and HTML versions of the pages, and stores them to the database. From these pages, the program identifies any links to other transient web pages/open architectures, recursively downloading and analyzing the content until all possible transient links have been found and captured. To date, this process has resulted in the capturing and storing of over eight million one hundred thousand tweets; one million three hundred thousand tweets URLs; one million two hundred thousand images; and forty‐eight thousand transient web pages. From the tweet metadata it can be determined that 69 percent of the total tweets are written in Arabic, 13 percent in English, and 18 percent are other or undefined. In a secondary step a Naive Bayes algorithm was applied to the transient web pages to determine the primary language and determined that 54 percent of the web pages are written in Arabic, 38 percent in English, and 8 percent are other or undefined.

### Analysis

**Figure 2:**
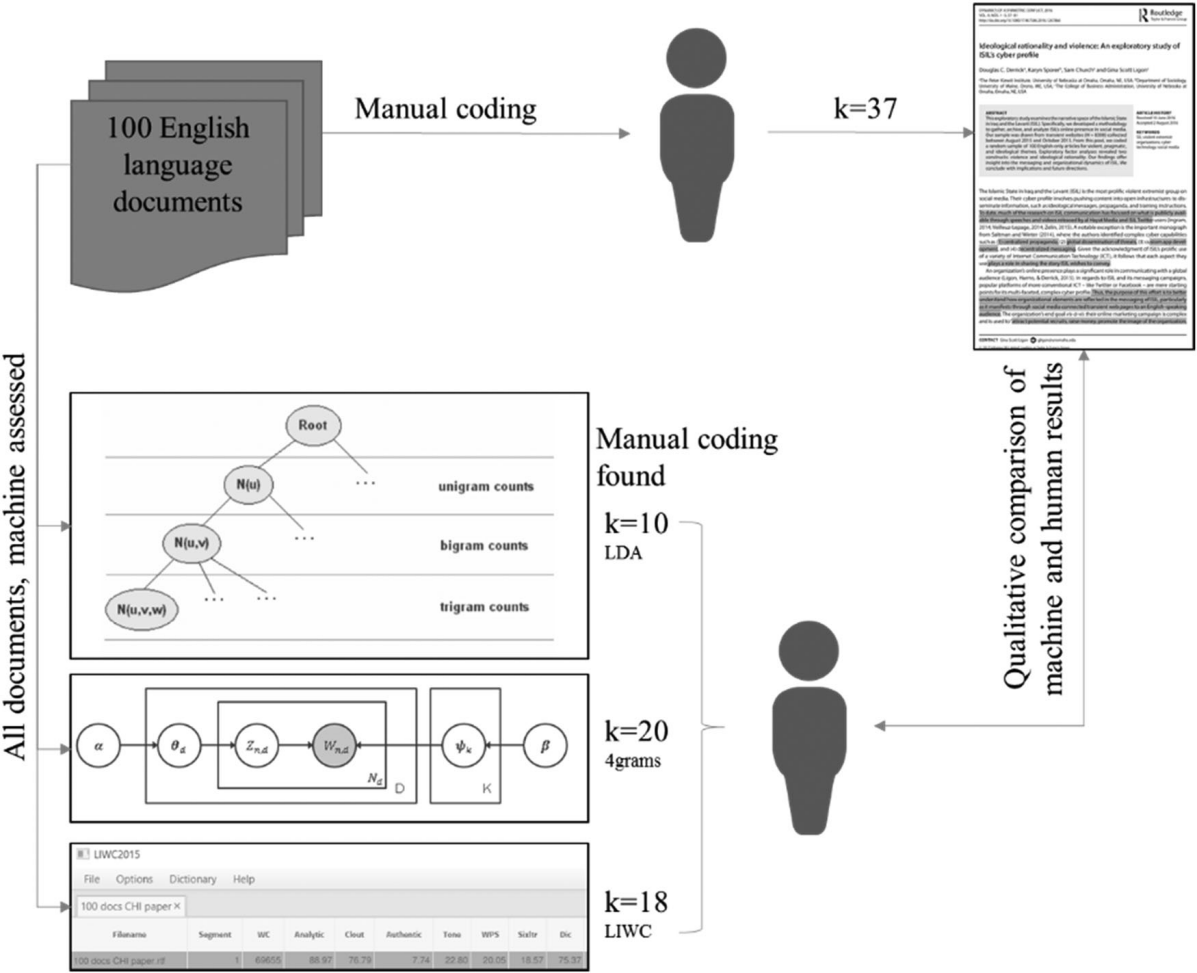
A High‐Level Workflow of the Current Comparative Study.

A high‐level overview of this article’s approach is as follows, and Figure 2 illustrates the workflow of the current study:

(1) Data Collection (see previous section, and Table 2).
  **Table 2:** Manually Coded Results of VEO Content Scraped From Open Architectures and Social Media (see Derrick et al., 2017b)

Quran	64	Help Locals	19
Legitimacy	43	Destroy property	19
Caliphate	38	Bayat	18
Education	37	Destroy by enemy	17
Violent items	35	Motivate	16
Mujahideen	34	Manuals	13
Apostate	34	Baghdad	12
Anti‐west	34	Shame	11
Jihad	31	Apocbattle	9
Sharia	30	Cyber	9
Destroy by ISIS	30	Hijrah	8
Atrocities	29	Hisbah	8
Mohammed	28	Repent	7
Media	28	Diyya	5
Justify	26	Ribat	5
Leader	25	Training	5
Territory	24	Village ldr	2
Military ops	24	Lone wolf	2
Shahid (Martyr)	20		

*Note*: The terms reflect the feature identified, and the numbers reflect the frequency the coder identified the item in the corpus.

ISIS, Islamic State in Iraq and Syria; VEO, violent extremist organization.
(2) Analyze text samples via standard text analytics toolkits.
(3) Name concepts found in machine results in order to compare with qualitative results (Table 2).
(4) Repeat steps four and five to produce range of accuracies and cross‐validate results.
(5) Compare results of automated processes with qualitative results.

Seven researchers trained in qualitative measurements of ISIS leadership and VEO communications evaluated the machine‐created features (explanatory characteristics) to measure goodness of fit to the human‐coded baseline in an open coding format. Intercoder agreement was assessed in a jury format where coders independently assessed the theme presented by the algorithm. Results were compared between coders, and disagreements were discussed with two or more coders and three faculty members with seven or more years of experience in qualitative research until one single concept was agreed upon (see Results). A significant problem in grounding truth between qualitative and quantitative results is in its assessment. Human and the text analytics results produced different numbers of themes (referred to as vectors where *k* ∈ {37, 10, 20, 18}). Due to the uneven vectors produced by the humans and algorithms (see discussion below) our study concentrated on qualitatively measuring goodness of fit. Finally, the themes were operationalized in four states: the theme was present, half present, partially present, or not present (see Tables 6 and 7).

The 2017 authors established thirty‐seven categories (themes) of activities and outreach taking place online, where the reported the Cohen’s *κ* interrater agreement as 0.64. These are thirty‐seven categories are represented in Table 1. By randomly selecting the one hundred web pages, the 2017 authors sampled a wide variety of types of web content produced by ISIS supporters as disseminated via transient open architecture, including pictures or photographs with short captions or headlines and text or essays. At the time of the data collection and analysis, English‐only web pages warranted deep empirical attention for three primary reasons. First, ISIS has directly engaged English speakers, particularly in the United States. For example, in 2014 ISIS established a “#AmessagefromISIStoUS” marketing campaign on Twitter and posted a YouTube video called “A message to the American People”—all in English. Second, given the threat of foreign terrorist fighters returning to the West, it is imperative to examine the messaging content that the majority of western citizens can read and understand. As such, despite the majority of ISIS propaganda being in Arabic or other non‐English languages, a deep understanding of ISIS’s messaging in English warrants attention. Finally, this is an exploratory study that can be used to benchmark and examine larger data sets with more languages.

The one hundred English‐only transient web pages provided a corpus comprising two hundred forty‐five thousand and seventy‐five words after removing transliterations (6areeq, shaam) and misspellings (th for the; viel for veil). Transliterations and misspellings have an outsized impact on result quality in the n‐grams analysis as they make the term matrix sparser, and are not recognized (and thus excluded) by the dictionaries in the LIWC analysis. As such, they were excluded for all three analyses. Hence, two hundred fifteen thousand four hundred and ninty‐nine words were recognized by English dictionaries (~88 percent). On average, 67 percent of the words from each transient website were detected as English. Words were transformed to lower case to avoid errors due to capitalization. Non‐alphanumeric characters were removed. Once a primary language of the webpage was determined, characters outside of the particular language were removed via regular expressions. URLs and other information specific to the hosting service were also stripped from the webpage content before the analyses.

The current study employed simple, off‐the‐shelf packages. This has several purposes. By employing standard packages, replicability is aided. Good practice demands employing the computationally simplest packages in terms of eventually benchmarking performance of more complex algorithms. Due to these reasons, we employ n‐grams and LDA (Blei et al., 2003; Jurafsky & Martin, 2014). Both algorithms are appropriate for creating feature vectors (vectors of explanatory characteristics) from unstructured text. While similar in concept, the two approaches are distinct.

The n‐grams are commonly used to summarize text given their basic co‐occurrence algorithmic basis. n‐grams can be trained and cross‐validated in a single corpus. This has the desirable property of being insensitive to—and thus comparably flexible with—unknown words. It has also been used in similar approaches in previous work (see e.g., Burnap & Williams, 2015, considering online hate). In the n‐gram model, each word is assumed to be drawn from the same term distribution. A topic is drawn if a predetermined number comprised of a string of words occurs. These strings are counted if the sequence occurs in the text 1+ times.

So, n‐grams are limited in contextualizing corpora as the results are context insensitive (Jurafsky & Martin, 2014). Therefore LDA is also implemented in this study (McCallum, 2002). A generative model, or a model with a joint probability distribution, LDA is even more recognized as fitting the task of modeling themes in text‐based corpora (Blei, 2012). A major draw, LDA’s bag‐of‐words assumption allows the context and not only the topics to be identified. It has been applied in similar detection scenarios, including detection of online pornography (Tang et al., 2009) and hate speech (Burnap & Williams, 2015). We follow the standard process of LDA with Gibbs sampling (Blei, 2012; Boecking, Hall, & Schneider, 2015; McCallum, 2002):

1. For each topic *k*
  i. Draw a distribution over words $\vec{\beta_{k}}\sim {\mathrm{Dir}}_{V}\left(\eta \right)$.
2. For each document *d*
  i. Draw a vector of topic proportions ${\vec{\theta }}_{d}\sim {\mathrm{Dir}}_{K}\left(\vec{\alpha }\right)$
  ii. For each word *n*,
    1. Draw a topic assignment $Z_{d,n}\sim \mathrm{Mult}\left({\vec{\theta }}_{d}\right)$, $Z_{d,n}\in \left\{1,\ldots ,K\right\}$
    2. Draw a word $W_{d,n}\sim \mathrm{Mult}\left({\vec{\beta }}_{Z_{d,n}}\right)$, $W_{d,n}\in \left\{1,\ldots ,V\right\}$

The hidden variables in this model are the topics $\vec{\beta }$, the proportions of topics per document $\vec{\theta }$, and the topic assignments per word Z. $\vec{\alpha }$ controls the concentrations of topics per document, while η controls the topic‐word concentrations. The multinomial parameters $\vec{\beta }$, the topics are smoothed by being drawn from a symmetric Dirichlet conditioned on the data.

Finally, the sentiment analysis package LIWC was employed to assess the primary structure of the content and latent meaning of the corpus. Meaning is fundamental in the case of radicalizing content, and Natural Language Processing testing and validation on VEO content (and other propaganda generally) is still lacking (Table 1). Sentiment analysis is generally more appropriate for tackling latent meaning (Chen, 2008; Liu, 2010; Mirani & Sasi, 2016). LIWC is a limitedly content‐sensitive sentiment analysis package (Pennebaker, Chung, Ireland, Gonzales, & Booth, 2007). This approach enables us to look not only at usage or context but also the latent meaning of the corpus (Pennebaker, Mehl, & Niederhoffer, 2003). The focus on latent meaning rather than individual wording has the secondary useful attribute of allowing assessment of context even in the case that the language/slogan changes, as the focus for LIWC is patterns in word usage, and not the term itself. While drawbacks and criticisms of LIWC’s approach exist (see e.g., Beasley & Mason, 2015; Panger, 2016), LIWC has been found to cope admirably well with relatively sparse social media data (Lindner, Hall, Niemeyer, & Caton, 2015), and has been successfully applied to suicide ideation scenarios (Handelman & Lester, 2007) and analyses of terrorist discourse (Cohen et al., 2016; Logan & Hall, 2019; Pennebaker, 2011). A large basis of literature employs LIWC scoring either directly or in combination with machine learning on social media data, making it the strongest benchmark sentiment analysis package to date (Coviello, Fowler, & Franceschetti, 2014; Hughes, Rowe, Batey, & Lee, 2012; Kramer, 2012; Lin & Qiu, 2013; Thelwall, Buckley, & Paltoglou, 2012; Thelwall, Buckley, Paltoglou, Cai, & Kappas, 2010).

## Evaluation

### n‐grams

**Figure 3:**
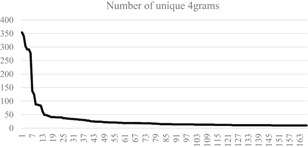
4gram Distribution by Term Volume Demonstrating Zipf’s Law.

Given the sparse corpus, we ran three experiments. The experiments looked at strings of three, four, and five words (or, *n*‐grams). Of these, 4grams produced the most compact results, reasonably preserving the grammar and structure of the documents. Instances of repetition or broken structure were consolidated when they were obviously related (i.e., United States Department of, States Department of State). This resulted in eight hundred and ninty‐seven unique observations producing five thousand three hundred and seventy‐one 4grams. Figure 3 shows that the distribution approximates Zipf’s Law. Twitter handles, tags, and IDs are removed.

In the manual assessment, the jury (a team of four researchers) found that ten topical themes emerged (Table 3). This is significantly lower than the thirty‐seven themes listed in Table 2. Approximately a third of the corpus involves self‐references to ISIS, and another quarter represents Quranic references to Islam and jihad. Fifty‐one instances of 4grams are unclassified as they are either internet jargon or otherwise defy meaningful interpretation in the 4gram form (e.g., “before right and after”; “singing” *n a n a* in the text).

**Table 3:** Results of the 4gram Analysis With Topic Categories

Topic	Comparison to Derrick et al. (2017b)	Example Text	*f*
Self‐reference	*Leadership; motivational speeches*	*compassionate islamic state wilayat: islamic state of iraq*	1,806, 33.6%
Religion	*Quran verses/teachings/prayers; Mohammed*	*may allah be pleased; the cause of allah; al fatawa volume 13*	1,399, 26%
US Government/Military	*Defense or justifications for violence; Military operations; cyberwarfare*	*defense information systems agency; state.gov department of state*	1,320, 24%
Delegitimizing Rival Leaders	*Diminishing the legitimacy of others; Apostate regimes*	*abu bakr al baghdadi; bahira omar yaqoob said; by jabhat al joulani*	262, 4.8%
Delegitimizing Rival Orgs	*Diminishing the legitimacy of others; destruction*	*gunfire exchange with rafida; the free syrian army*	156, 2.9%
Transliteration		*of ahrar al shaam; in deir az zour*	105, 1.9%
Unity	*Apostate regimes*	*it saddens me how; brothers in the west*	103, 1.9%
Public Institutions		*national institutes of health; army corps of engineers*	81, 1.5%
Society	*Media centers*	*may contain sensitive material; islamic states reports src*	88, 1.6%
Misc		*n a n a*	51, .09%

*Note*: Column 1 represents the contained topics; column 2 compares to Derrick et al. (2017b); column 3 contains sample 4grams; column 4 indicates the frequency of occurrence and percent of corpus represented. The long tail is not included, thus the total does not equal one hundred percent.

In comparison to the original study, the 4gram results are less granular than the human coders. What is compelling about these findings is that while n‐grams provided fewer categories than human coders, the observations within these categories resulted in higher‐level themes when compared with human coders—in other words, 4grams produced more compact results compared with human coders. Table 7 demonstrates that thirteen of the themes found in (Derrick et al., 2017b) were subsumed by the seven n‐grams themes identified in Table 3. With one thousand three hundred and twenty unique 4grams, our results indicate that the presence of discourse about the US government and military is much more dominant than the qualitative coders initially assessed in Derrick et al. (2017b). This may be due to the fact that the qualitative coders added references to the US government and military into adversarial themes. Interesting themes also emerged from the n‐gram results that were not identified by human coders, especially transliterated aspects and broad references to western society. Unity, a theme identified 88 times by n‐gram, is particularly of interest as it theoretically should be tied to reasons for joining terrorist organizations (i.e., Horgan’s work on “opportunities for camaraderie” indicates that terrorist propaganda is replete with messages about kinship and unity [Derrick et al., 2017b]). As social media are one of the major recruitment arms of ISIS and VEO groups broadly speaking, this particular theme would have been expected to be much more present in the themes identified qualitatively.

Overall, naïve approaches like n‐grams deliver visibility into the corpus, but poorly approximate qualitative benchmarks, particularly community‐oriented topics and the least‐common results found manually (i.e., the long tail). This is likely due to the word‐counting approach used. With its higher granularity, n‐gram features miss the nuance and detail of qualitative results across all categories, but do detect subtleties that human coders missed in other cases.

### LDA

The MAchine Learning for LanguagE Toolkit’s (MALLET; McCallum 2002) implementation of LDA was used to create feature vectors in the style of Boecking et al. (2015). Several text‐preprocessing steps were conducted. Unwanted characters and items in the messages are removed such as punctuation, emoticons, user‐mentions, and URLs. Common stop words are also removed. A single input document of the transient pages is created (*n *= 245,075) and passed on to LDA.

**Figure 4:**
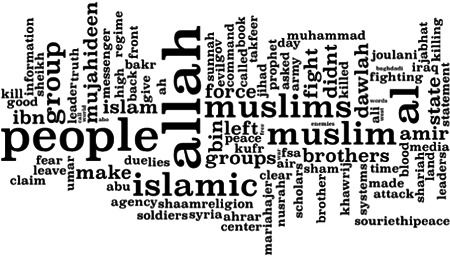
Word Cloud Representing the One Hundred Most Frequently Returned Terms in Twenty Feature Vectors of Jihadi Discourse.

We used a symmetric Dirichlet distribution, initializing *α* to 0.5; β corresponds to the word distribution for each topic (here, 15). *K* represents the number of distinct concepts. We estimated the number of concepts in the corpus by examining if the resulting topic models of different sizes resulted in reasonable, cohesive topics that are not included as training data. All reported results are subject to a 10‐fold cross‐validation. Thus, 2 × 6 experiments were conducted: one treatment where the corpus contained twitter handles, and one where the corpus did not. No significant differences were observed. We conducted two hundred runs apiece for each *K* ∈ {10, 15, 20, 25, 30, 37} on the training set (90 percent of documents), and measured the perplexity on the validation set (the remaining 10 percent). Of these, *K* = 20 yielded the best results. This is likely due to performance degradation from the sparse text. Figure 4 represents the one hundred most frequent terms where the word size is proportionate to its probability in the topic, but not proportionate to its frequency in the corpus.

The twenty features that emerged converge more closely to the qualitative results in terms of the focus on religious violence and apocalyptic viewpoints (Table 4). This lines up with the two primary themes of ISIS content identified in Derrick et al. (2017b), namely, Ideological Rationality and Violence (Table 5). Particularly interesting is that only two Twitter handles, @almoohajeermos and @souriethipeace, were allocated in the experimental results with Twitter data. This indicates the intensity of these two handles’ posts on specific topics. Surprisingly, the LDA features highlight the hotly fought‐over cities Homs, Aleppo, and Mosul, but neither Raqqa (the then‐capital of ISIS) nor Baghdad (as indicated in Table 2) appear. Also broadly absent are features discussing the community around ISIS, something quite present in the qualitative results. In agreement with the 4gram results, religious outreach is hugely present. However, the US military, civil society, and public media are nonexistent. Overall, however, LDA outperformed n‐gram in that it identified seventeen of the constructs found by human coders (see Table 7). Also, LDA was more effective at identifying violent themes. Given its relatively low computational complexity and the fact that it is not strictly keyword or phrase‐based, LDA may be useful for quickly identifying text‐based content that violates Terms of Service, in terms of violent content.

**Table 4:** Results of the Latent Dirichlet Allocation (LDA) Analysis With Topic Categories. Column 1 Represents the Named Topics; Column 2 Contains Sample Topics

Comparison to Results of Manual Coding in Benchmark Article (Derrick et al., 2017b)	Text in Feature Topics, *α* = 0.5, *β* = 15, *K* = 20
Caliphate/Unity	Fight dawlah syria shariah war mentioned back path tawheed called banner allaah evidence council benefit
Sharia Law/Motivational Speeches	Group muslim fighting clear center men matter support dont disbelief reality areas true sharia worship
Military Operations/Caliphate	Muslims left person making killed caliphate believers apostate proof makes mariahajer face fighter haqq homs
Delegitimizing Rivals/Media	People time good ali god taliban permissible end amir wal didnt twitter giving pray deeds
Military Operations/Atrocities	Make leave kill sham asked regime law wa put khawarij khilafah video judgement battalion fact
Anti‐West	Islam systems information ibn agency takfeer found world great defense agreed prison means abdul albani
Delegitimizing Rivals/Leadership	People army groups disbelievers fought quran evil hanisibu free syrian hadith imam based allegiance stop
Violence	State islamic shaam iraq ahrar gov join attacked photo attacks difficult munafiqeen defected hour turn
Jihad/Mujahedeen	Jihad mujahideen religion leaders soldiers west part attack wealth taking kuffar followers doesnt control find
Mujahedeen/Bayat	Killing kufr air base today battle ziadzd accept women states lot muhajireen camp sake bayah
Delegitimizing Rivals	Abu al command nusra told death signal lol sin shaykh mujahid members jn muhajir operations
Quran Verses, Teachings, Prayers/Apostate Regimes	Allah prophet messenger peace sunnah blessings pleased heard merciful judge revealed family barracks surely christian
Military Operations	Khawrij fsa blood day scholars knowledge brother claim city military mosul message amir till commander
Delegitimizing Rivals/Apostate Regimes	Ummah weapons muhammad call rule land apostates years dead aleppo mullah knew omar long mariahajer
Military Operations/Motivational Speeches	Made killed souriethipeace abo words allies hasan almoohajeermos fighters back happened night jabalalaiza actions intelligence
Quran Verses, Teachings, Prayers	Allah truth give lies book high life volume enemies lying mercy fatawa lack speech hand
Anti‐West/Shaming	Force brothers media ruling reason place order office son lie sisters hell town issue witness
Self‐Reference	Man due allah laws fear isis victory news leadership emirate false matters country live praise
Delegitimizing Rivals	Al jabhat nusrah front joulani statement deir zour az started qaidah jawlani central approach joined
Leadership	Al sheikh bin ah leader umar bakr shari baghdadi ayman abdullah zawahiri liwa abi speaking

**Table 5:** Component Transformation Matrix Results of the Exploratory Factor Analysis (EFA) on Linguistic Inquiry and Word Count (LIWC) Sentiment Analysis Data

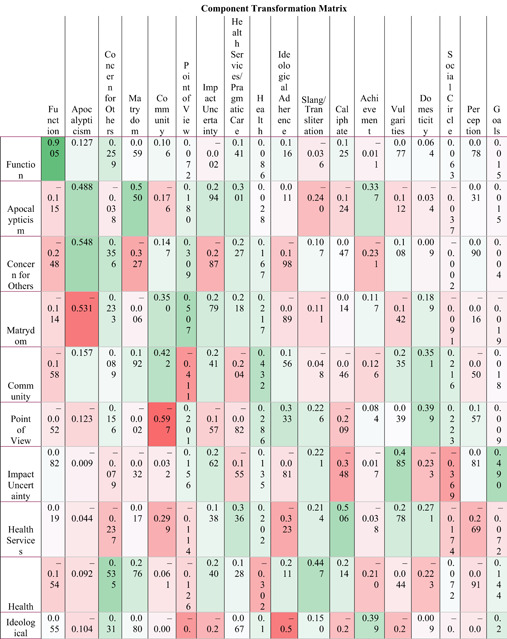 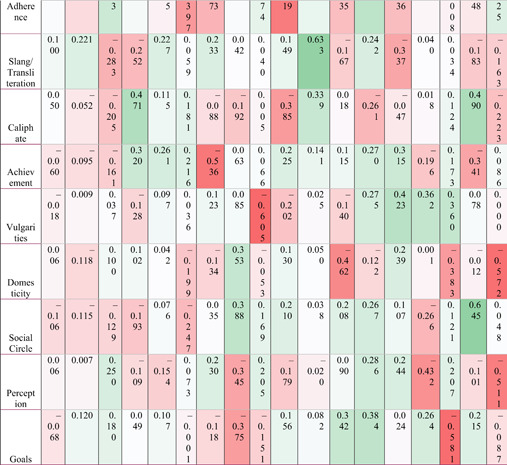

### LIWC

Finally, we assessed the latent structure of the sentiment behind the text with LIWC (Tausczik & Pennebaker, 2010). Considering composite variables, LIWC 2015 identified that 90.41 percent of the text could be classified as “Analytical,” indicating the text’s propensity to portray reasoning: formal, logical, and hierarchical thinking patterns. Analytical texts might be perceived as intellectual, rational, systematic, emotionless, or impersonal. On the other hand, the analysis found that only one percent of the corpus could be described as having an “Authentic” tone. Authenticity in the case of LIWC 2015 is closely related to deception. Both values are largely outside of their expected ranges as established in Pennebaker, Boyd, Jordan, and Blackburn (2015). These aspects suggest that the text is pitching a vision of the Caliphate that aims to recruit individuals, albeit in a way that doesn’t reflect the on‐the‐ground reality.

The two hundred fifteen thousand four hundred and ninty‐nine words recognized by English dictionaries from the one hundred web pages are scored for sentiment over sixty‐four psycholinguistic categories. With this numeric data, we assess the latent structure of the discourse with an exploratory factor analysis (EFA) run in SPSS Statistics 24. EFA allows us to understand how the concepts and the emotions behind the corpus combine, creating a more granular assessment of online jihadists. The EFA employed a varimax rotation, which converged in twenty‐one iterations, suppressing coefficients below 0.3. The EFA produced eighteen latent constructs (Table 5) based on the sentiment of the text, which were named by the content experts as discussed above. Table 5 displays the component transformation matrix (CTM). The CTM transforms rotated loadings by postmultiplying the matrix of the original loadings by the transformation matrix. The values in the CTM are functions of the angle(s) of rotation of the components. As they are measures of rotation degree, the intersection of two constructs is not expected to equal 1 as in a correlation matrix (For more information on EFA and CTM, please see Harman, 1976). For the 18 × 64 rotated component matrix listing the loading factors, please see the Supplementary Materials online.

Similar to LDA, LIWC directly matched human coders on 16 constructs, and LIWC’s results lined up with those most frequently identified by humans (e.g., violent content; see Tables 6 and 7 for more detail). LIWC also identified groupings that the human coders did not (e.g., ideological adherence, as discussed in Derrick et al. 2017b). Moreover, the two primary themes of ISIS content identified in the original article are strongly present in the LIWC results (see Supplementary Materials; Table 7). The alignment of the principle component analysis (PCA) results with the previous findings suggest that the LIWC constructs demonstrate construct validity for VEO research in as far as it concerns social data. Additionally, LIWC identified aspects of communal living not present in the other feature vectors, similar to the Unity theme found by the 4grams analysis. Thus, while LIWC was not a perfect match to the human coder, it was a close approximate on the most important constructs. LIWC particularly identified subtler groupings that human coders also found, which were however lost in the 4gram and LDA results (e.g., impact uncertainty).

**Table 6:** n‐grams, LDA, and LIWC Results Compared With the Two Major Themes of Ideological Rationality and Violence Reported in Derrick et al. (2017b)

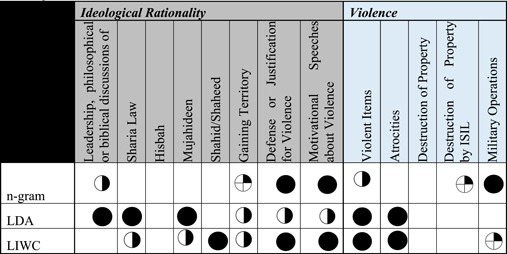

*Note*: Fully darkened circles indicate fully present; half indicates mostly present; a quarter circle indicates partially present; an empty space indicates not present in the results.

**Table 7:** Full Comparison of n‐grams, LDA, and LIWC Against Manually Coded Radicalizing Discourse Found in Transient Web Pages. Fully Darkened Circles Indicate Fully Present; Half Indicates Mostly Present; A Quarter Circle Indicates Partially Present; An Empty Space Indicates Not Present in the Results

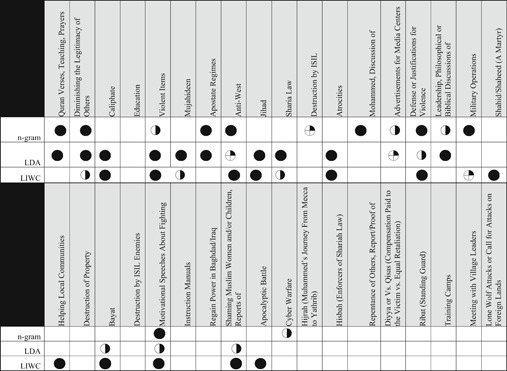

## Discussion

Overall, all three automated coding procedures performed similarly to the human coder in terms of detecting the most frequently occurring content (e.g., anti‐Western sentiment). Their performance varied wildly from the 2017 results at the long tail. Table 7 compares the efficacy of each computational approach as compared to the human coders. While no one procedure matched human coder findings perfectly, human‐identified themes such as Qur’anic Verses, Diminishing the Legitimacy of Rivals, and Anti‐West rhetoric were identified by n‐gram, LDA, and LIWC. However, less‐frequently occurring themes identified by human coders (e.g., Hisbah,^3^ Training Camps) were not detected by any of the automated techniques. This has several implications. It is reasonable to assume that automated processes can be powered by off‐the‐shelf text analytics packages to detect and classify major themes; however, we cannot expect a high overall accuracy rate due to these approaches’ lack of nuanced or latent theme detection. This split between effectiveness when classifying common themes and latent or nuanced themes has been shown in practice by those platforms that publish their results (e.g., Facebook).

While it is encouraging to find that humans and machines recognize and understand major concepts similarly, it is disappointing that the machines did not detect themes like Lone Wolf/Call for Attacks on Foreign Lands, as these are the type(s) of propaganda which can be acted on with very little interaction between the group and the individual (Ligon et al., 2018), and thus are harder to practically manage. The reasons for the lack of signal at the long tail could be many, in addition to high signal/noise problems. Some of the text may be accompanied by images, which are out of scope for this study. With the original study’s *α* of 0.64, it is possible that the human classifiers artificially compartmentalized the structure of the text compared to computational classification (e.g., Hijrah could belong to Qu’ranic Verses/Teachings).

## Conclusion

Much like pornography, “you know VEO content when you see it”—describing it however is not straight‐forward. Where the qualitative efforts are valuable, their practical usefulness is limited due to the nature and scale of the content, as well as with inherent bias added by human processing. Automated classification based on latent patterns in radical content promises to be more efficient. This work finds however that the computational approach has not yet fully approximated manual coders’ understanding of ground truth. Reasons for this are varied, but are likely due to the differences in humans’ holistic consumption of VEO content compared with the reliance of computational approaches on naïve pattern mining of the data. While neither approach is perfect, a computational approach that misses highly emotive VEO content (e.g., Lone Wolves) is not sufficient to implement in a real‐world scenario. This has been practically experienced by open social platforms as they reactively manage and remove VEO content. This work is a first effort toward empirically outlining possible reasons that misclassifications can (and do) occur. On the basis of these results, we argue that before fully automated approaches can be implemented, a more careful blending of human content understanding and machine abilities is required.

In response to the original research question of how well the processes approximate one another, it is compelling and promising that many high‐level concepts transfer well, especially themes surrounding violence and delegitimizing rivals. However, the granularity found by human coders is absent, for example, the machine approaches missed themes of education, instructional materials, and some theological topics like Hisbah. And indeed, some concepts like Unity (found by the n‐gram analysis) were not present in the 2017 results. This suggests that while there is more work to be done, a computationally efficient and theoretically‐grounded set of features can be developed to aid in automatically detecting radical content on the open internet. The missing puzzle pieces are described in the coming section.

## Limitations and Future Work

Despite the global outlook of jihadists, the lingua franca of groups like ISIS is Arabic, which is poorly covered in the literature. This is important to note for any language‐based automated detection task, as it has been shown that prediction accuracy is best when the locally dominant language is also employed in conjunction with English (Boecking et al., 2015). Thus, a serious limitation to our approach is the lack of availability of validated Arabic NLP packages. Currently available packages like CORE NLP and Weka only offer partial functionality; LIWC’s Arabic dictionaries are also still in the validation process. Our approach also lacks several more expansive or more granular assessments due to the lack of reliable Arabic language algorithms. This also partially explains the relatively simplistic, off‐the‐shelf approach selected. Our approach relies on character recognition to split multilingual texts; this unsophisticated approach would be better treated with language detection capabilities and could have possibly caused misallocation of words. Other approaches for text processing including *td‐idf*, entity recognition algorithms, or lemmatization do not fit the current scope, although they are being considered for future work. *tf‐idf*, a process to establish how important a word is in a given document, was not included in the initial work as it may inadvertently misallocate words that appear frequently (e.g., self‐references, references to religious figures), which are pertinent to ISIS’s branding and recruitment strategies (Derrick et al., 2017b; Ingram, 2016).

Future works may also consider evaluating the accuracy of the assignment of documents to specific topics, and not merely the creation of topics as demonstrated here. Finally and possibly crucially, some web pages are accompanied by images; this could explain the granularity of the human‐coder results, particularly at the long tail, which is not replicated by text analysis algorithms employed in the current analysis. More work needs to be directed at the analysis of images (pictures and videos) alone and concurrently with text‐based feature vectors.

Possible extensions of this work are many. The foremost goal is developing a flexible, detection algorithm to support the classification of radicalizing content. This can include language‐based features, metadata‐based features (views, shares, likes), or image properties. Such content requires that any feature‐based classifier be linguistically localized, and extensive work must be done on Arabic NLP capabilities to realize this. Another extension is understanding precisely what content radicalizes. This requires a multi‐method approach, including content analysis, topic modeling, and sentiment analysis. To support triangulation of the above results, an eye‐tracking study evaluating what content is consumed by reviewers when they view VEO content would be useful. At this point, we can expect to find an approximation of the “ground truth,” which allows us to begin working on predication scenarios.

Validating our approach across different open and social media platforms would help understand the context of radicalization, and pathways of radicalization of (vulnerable) individuals. Finally, from both the perspectives of content generation and research, the next frontier of social media research is in visual media. Computer vision has been employed recently to combat human trafficking (Dubrawski et al., 2015) and child pornography (Ulges & Stahl, 2011) but has yet to be trained and tested on jihadi content. Research is required to adapt the concepts to the VEO scenario, and expand the capabilities of the computer vision domain by training algorithms on radicalizing content scraped from outlets like YouTube, Instagram, and visual content from sites like Twitter and Facebook.

## Supporting information

Additional Supporting Information may be found in the online version of this article at the publisher’s website.

Supporting information

## Notes

1 “Leadership decision making” refers to the strategic, operational, and tactical decisions made by violent extremist leaders to ensure the survival of their organization. For instance, leadership decisions on how to brand their organization play a large role in the specific types of people they can recruit.

2 At the time, the Chechen Mujahidin would qualify as a VEO since the organization showed markers of operational collaboration, specialization, and a shared ideological mission against Russian occupation (Ligon et al., 2013).

3 Hisbah refers to the individual and collective duty to intervene and coercively “enjoy good and forbid wrong” in accordance with Sharia law.
