# Supplementary material for: Do Machines Replicate Humans? Toward a Unified Understanding of Radicalizing Content on the Open Social Web
Source: Policy Internet. 2019 Sep 26;12(1):109–38. doi: 10.1002/poi3.223 (PMC10647225; doi:10.1002/poi3.223)
Supplement: Supplementary file 1 — Supporting information [file POI3-12-109-s001.pdf]

Rotated Component Matrix[a]

	Component																	
	Function	Apocalypticism	Concern for Others	Matrydom	Community	Point of View	Impact Uncertainty	Health Services/ Pragmatic Care	Health	Ideological Adherence	Slang/ Transliteration	Caliphate	Achievement	Vulgarities	Domesticity	Social Circle	Perception	Goals
funct	0.853	0.131	0.153	0.342		0.107		0.136					0.124					
pronoun	0.887		0.205							0.111		0.298				0.155		
ppron	0.735		0.226							0.221		0.448	-0.128			0.244		
i	0.339			-0.139				0.162				0.764	-0.100					
we	0.268						-0.136			0.125					0.181	0.778		0.116
you	0.436			0.102		0.287				0.716			-0.161					
shehe	0.579			0.205		-0.110	-0.230			-0.121		0.451			0.100	-0.299		0.207
they	0.563	0.177	0.464												-0.130	0.340		-0.230
ipron	0.904		0.126												-0.149			
article	-0.136			0.525			0.139				-0.203		0.238	-0.211	-0.173	-0.240		0.108
verb	0.904		0.152					0.194				0.182		0.149				
auxverb	0.929													0.157				
past	0.574					-0.124	-0.172	0.370		-0.210		0.501						
present	0.838		0.202			0.181				0.169				0.141				-0.179
future	0.727					0.102	0.119			0.117		-0.256		0.204				0.334
adverb	0.796		0.101	-0.137				0.287				0.267						-0.181
preps	0.369	0.421		0.551		0.309	0.111	0.229		-0.119	-0.121		0.266	-0.108				
conj	0.929		0.147									-0.117						
negate	0.791					-0.106				0.305		0.122	-0.143		0.165			-0.105
quant		-0.122	0.103	0.220	0.311		0.678			-0.161						-0.147		
number	-0.347			-0.429	-0.258	-0.187	-0.313	-0.142					-0.211	0.108		-0.198	-0.181	
swear	0.138						-0.103							0.830				0.117
social	0.662	0.162	0.253	0.232	0.309					0.210		0.274	-0.103			0.251		
family					0.782				0.224	0.178		0.126	-0.136			0.248	-0.133	-0.158
friend	0.321		0.179	0.162		-0.202	-0.391	-0.125	0.250	-0.160		-0.101	0.104		0.241		0.127	0.238
humans	0.166		0.156	-0.151	0.818								-0.104					
affect	0.385	0.697	0.252	0.274		-0.146				0.300							0.139	
posemo	0.373			0.546	-0.101	-0.234				0.492				0.124				
negemo	0.209	0.896	0.251					0.101									0.116	
anx	0.245		0.759		0.108			0.166		0.114	0.125							0.142
anger		0.962																
sad	0.169		0.683		0.159		-0.227	0.306	0.108			-0.146			0.241		0.135	
cogmech	0.880		0.128	0.154			0.108						0.152				0.211	
insight	0.647			-0.131		0.224	-0.170					0.108	0.261	-0.131	-0.131	-0.265		
cause	0.615			-0.368				0.171					0.231		-0.167		0.336	
discrep	0.766			-0.160			0.190		0.113	0.130					0.149		0.124	0.171
tentat	0.187			0.273		-0.119	0.769						0.127		0.142			0.111
certain	0.370				0.763		0.163		-0.135									
inhib	0.270					-0.114	-0.113			0.751			0.230		-0.110	0.100		
incl	0.713		0.242	0.141			-0.221		0.185	-0.176		-0.221		-0.104		0.167	0.285	
excl	0.852									0.281			-0.103		0.145			
percept	0.282		0.331			0.792							-0.116				0.277	
see						0.871	-0.103	0.183									0.124	
hear	0.461		0.654			0.212			0.116			0.223	-0.165		0.107			0.104
feel						0.265		-0.103					-0.103				0.838	
bio	0.223		0.144				0.125		0.841					0.244	0.274			
body	0.281	0.108	0.304	-0.178	0.138		0.254		0.313	0.118		-0.134	-0.128	0.259		-0.226	0.173	0.458
health									0.946									
sexual	0.160	0.123										0.145		0.848				
ingest	0.144								0.140						0.841			-0.239
relativ		0.352		0.334		0.372	0.218	0.649					0.220					
motion	0.436		0.307				-0.118	0.674				0.114						
space	-0.170	0.384		0.361	-0.126	0.448	0.197	0.273				-0.102	0.315	-0.104				
time	0.277	0.198	0.168	0.116		0.223		0.720				0.162	-0.137					
work	-0.267		-0.151		-0.151		0.379						0.674		0.148		-0.132	
achieve	0.225			0.179	-0.114							-0.168	0.687					0.104
leisure	-0.132	-0.149		-0.118	0.551	0.332	-0.123	-0.162					0.371		-0.119	-0.149	-0.213	-0.133
home			0.276	-0.227		0.129		0.134	0.135				0.198		0.654	0.276		
money	0.133														0.276	-0.124	0.145	-0.761
relig			-0.123	0.721	-0.154	-0.218	0.252	0.102						0.217		0.154		
death		0.876								-0.114							-0.140	
assent											0.980							
nonfl											0.980							
filler	0.351		0.644	-0.106		0.165	0.222								-0.202		-0.222	

Extraction Method: Principal Component Analysis.

a. Rotation converged in 21 iterations.
